# Serious Right Coronary Artery Thrombosis Revealing Behçet’s Disease in a Female Patient: A Case Report

**DOI:** 10.7759/cureus.11382

**Published:** 2020-11-08

**Authors:** Fadoua Mouedder, Karima Benbouchta, Nabila Ismaili, Noha Elouafi

**Affiliations:** 1 Cardiology, Mohammed VI University Hospital, Oujda, MAR; 2 Cardiology, Mohammed I University, Epidemiological Laboratory of Clinical Research and Public Health, Oujda, MAR; 3 Cardiology, Mohammed I University, Oujda, MAR

**Keywords:** st-segment elevation, right coronary artery, coronary thrombosis, behçet disease, cardiac involvement

## Abstract

Although atherosclerosis remains the major cause of acute coronary syndrome, there are many other etiologies that should be taken into account, especially in young patients with no atherosclerotic risk factors. Coronary involvement is extremely rare in patients with Behçet's disease, notably in young patients. In addition, acute inferior myocardial infarction revealing Behçet's disease has rarely been reported. Through this article, we report a case of Behçet's disease with arterial involvement diagnosed after myocardial infarction resulting from thrombosis of the right coronary artery in a 50-year-old woman with no specific medical history.

## Introduction

 Behçet's disease (BD) is a systemic vasculitis of unknown etiology, characterized by remitting and relapsing episodes of genital and oral ulcers, ocular lesions, and a number of systemic manifestations [1]. Vascular involvement in BD can involve both veins and arteries of any diameter. Venous involvement in BD is more frequent than arterial involvement; the latter can be life-threatening and mainly concerns large arteries but also can affect the peripheral arteries. Coronary artery disease is very rare in BD and poses several challenges. We report a female patient diagnosed with BD who presented with acute coronary syndrome caused by right coronary artery thrombosis.

## Case presentation

**Figure 1:**
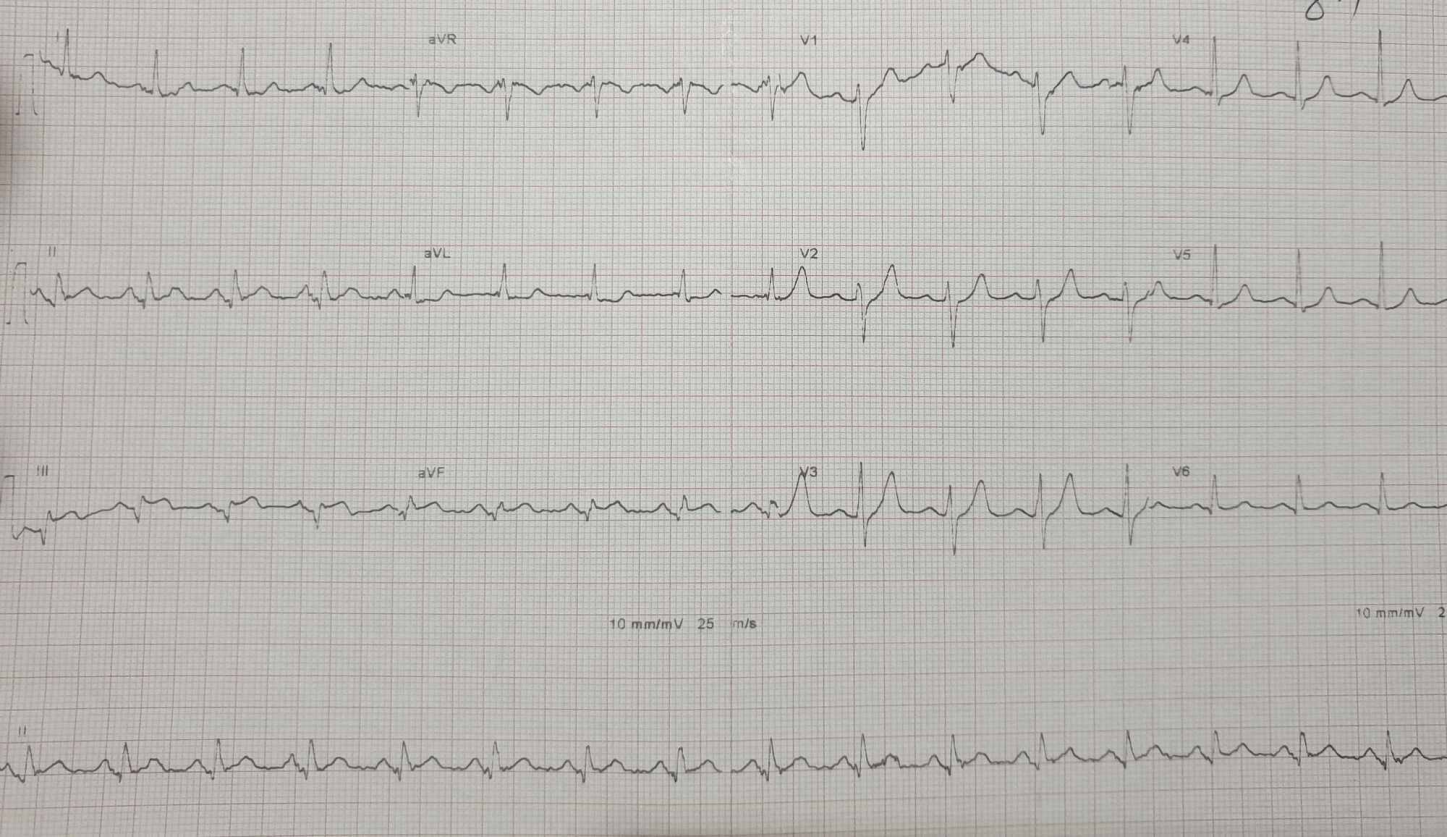
ECG at the sixth hour showing ST-segment elevation in inferior leads

We report the case of a 50-year-old woman with a four-year history of bronchiectasis treated with salbutamol, with no known major risk factors for atherosclerosis causation. This patient presented to the emergency department with acute and constrictive chest pain evolving for the last six hours. Physical examination noted stable hemodynamic status without signs of heart failure. ECG showed ST-segment elevation in inferior leads (Figure 1).

**Figure 2:**
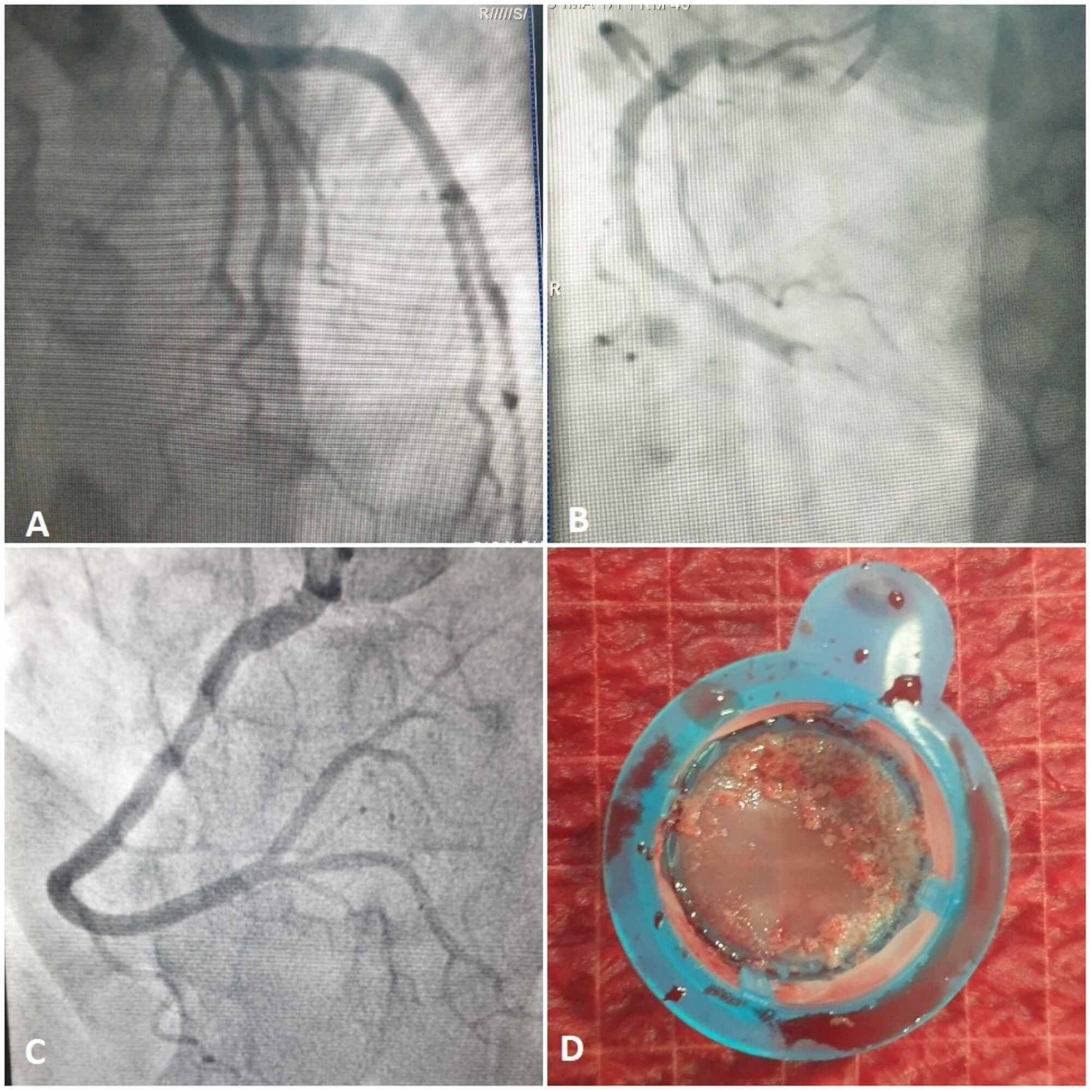
Coronary angiography at the acute phase of myocardial infarction a: Angiographically normal left coronary system b: Distal right coronary artery thrombosis with TIMI 0 flow c, d: Thrombus aspiration retrieving red thrombi restoring TIMI 3 flow TIMI - thrombolysis in myocardial infarction

The diagnosis of a non-complicated inferior ST-segment elevation myocardial infarction (STEMI) seen at the sixth hour was made. Given the resolution of chest pain, the patient did not benefit from thrombolytic therapy. Given the fact that primary angioplasty was not available at that hour, treatment with antithrombotics was started combining aspirin, clopidogrel, and enoxaparin. Echocardiography revealed extended akinesis of the inferior and inferoseptal walls and hypokinesia of the inferolateral wall, without intraventricular thrombus. Left ventricle ejection fraction was estimated about 45% without mechanical complications. At the tenth hour, the patient underwent coronary angiography, which showed a fresh thrombus floating in the distal part of the right coronary artery with thrombolysis in myocardial infarction (TIMI) 0 flow and angiographically normal left coronary system. Thrombus aspiration using a catheter was performed, retrieving red thrombi with the restoration of TIMI 3 flow without placement of a stent (Figure 2).

The patient was kept on antiglycoprotein IIb-IIIa (anti-GpIIb-IIIa) for 24 hours.

**Figure 3:**
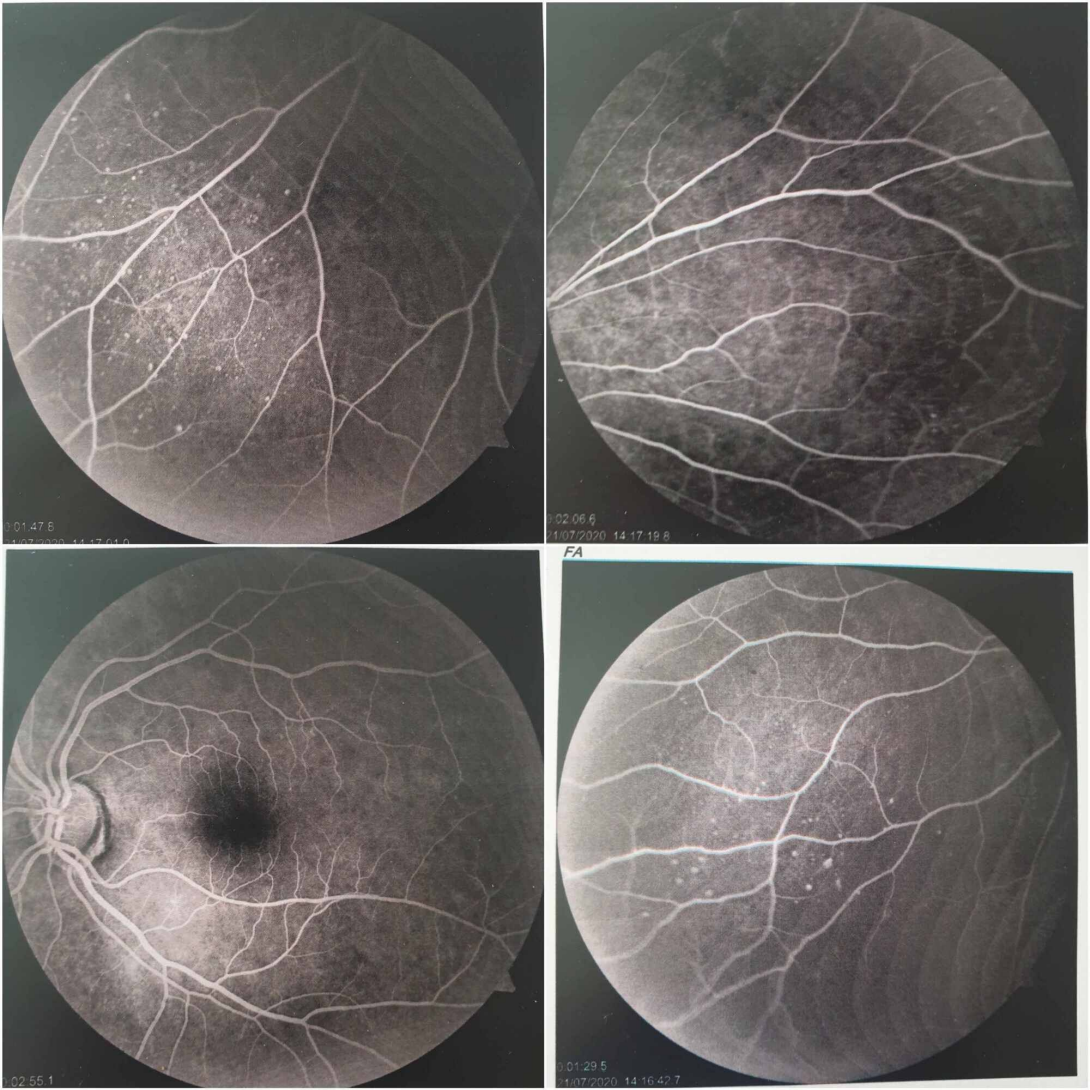
Fluorescein angiography showing signs of vasculitis and microaneurysms suggestive of Behçet's disease

The etiological investigation did not reveal other atherothrombosis risk factors. Given the important thrombotic burden in the right coronary artery associated with angiographically normal coronary arteries, thrombophilia was highly suspected. Screening for anticardiolipin antibodies, hyperhomocysteinemia, antithrombin, and protein S and C deficiencies were negative. After careful questioning, the patient revealed a history of arthralgias and recurrent buccal and genital ulcers. Physical examination revealed a buccal ulcer and a vaginal scar that would be compatible with a vaginal ulcer sequel, which were highly evocative of BD. Fundoscopy showed signs of uveitis. Fluorescein angiography showed signs of vasculitis and microaneurysms suggestive of BD (Figure 3).

**Figure 4:**
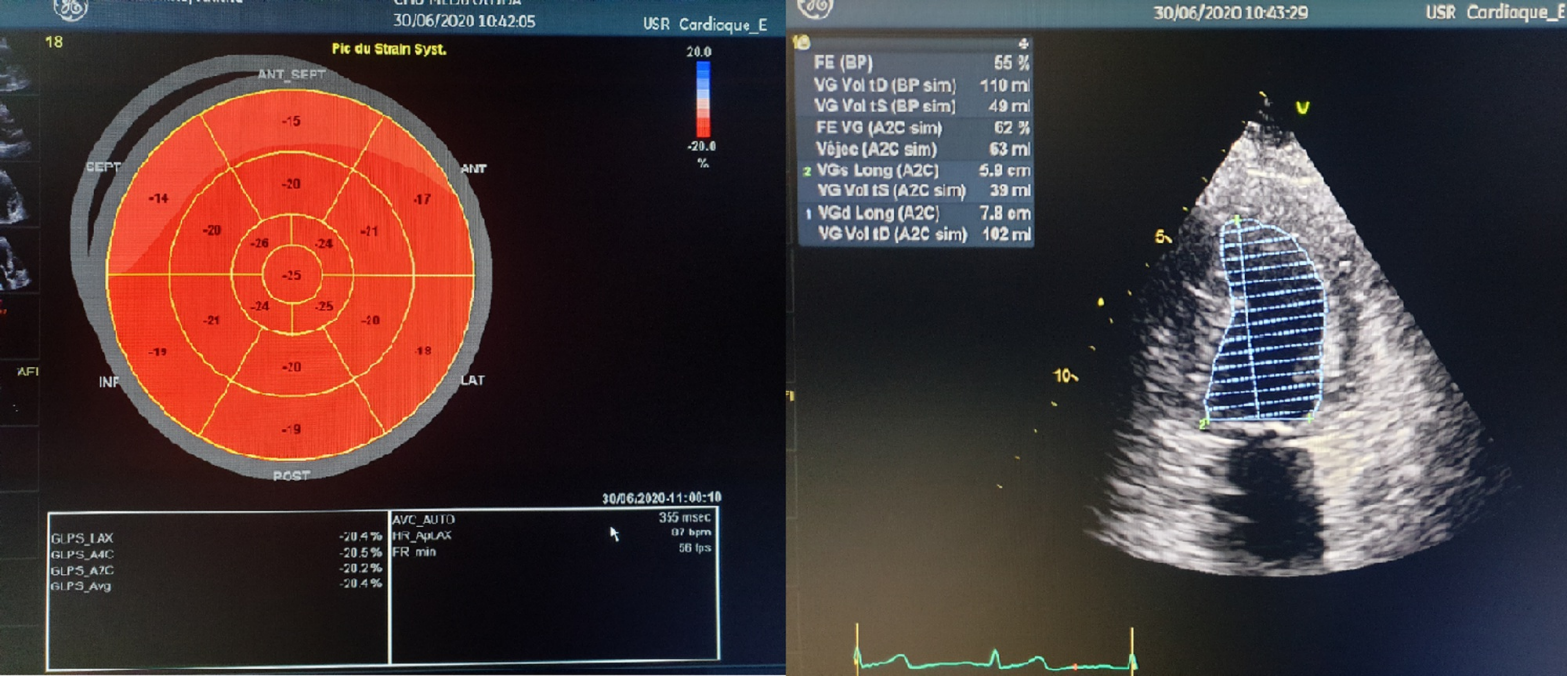
Echocardiography showing recovery of normal LVEF LVEF - left ventricular ejection fraction

Based on the international criteria for BD, the diagnosis of angio-Behçet was retained. As a consequence, the patient received dual antiplatelet therapy (acetylsalicylate and clopidogrel), antivitamin K (Sintrom®), bisoprolol, ramipril, and atorvastatin. The clinical course remained uneventful, and echocardiography repeated a month later showed improved left ventricular ejection fraction at 55% (Figure 4).

## Discussion

​​​​​BD is a systemic vasculitis of unknown etiology. It is more common in young adults from the Middle East and Mediterranean rim and affects twice as many men as women. Its diagnosis is clinical, based on international criteria that have high sensitivity [1, 2].

BD is characterized by remitting and relapsing episodes of oral ulcers, genital ulcers, skin, and ocular lesions. Many systems may be involved, such as neurological, musculoskeletal, and cardiovascular systems [3]. Cardiovascular involvement is rare but can have different manifestations and can be life-threatening. According to several studies, the prevalence of cardiovascular involvement is around 29% [4].

Valvular disorder, intraventricular thrombosis, and coronary arteries aneurysms are the most reported cardiac manifestations in BD, but there are other disorders such as myocarditis, pericarditis, endocarditis, and conductive tissue disorders [5].

The vascular tropism of BD is manifested by phlebitis, which is a major sign of the disease [6]. Arterial involvement is rare compared to venous involvement. Arterial involvement makes up only 20% of vascular complications [7]. Arteries of any size may be affected resulting from either occlusion or an aneurysm. The major involved arteries are pulmonary, femoral, popliteal, subclavian, and carotid arteries. Coronary involvement is extremely rare, especially in young patients [8].

The majority of cases described in the literature, which presented with coronary events, were previously diagnosed with BD, and followed regular treatment. Less frequently, coronary complications can occur as the first manifestation of this disease, which was our patient's case.

Coronary artery disease is very rare in BD but poses several diagnostic and therapeutic challenges and can be life-threatening. To our knowledge, there are three forms of coronary artery involvement during Behcet's disease: pseudoaneurysms, stenosis, and thrombosis [9].

The exact cause of acute coronary syndrome in patients with BD is not clearly understood. Unlike other inflammatory diseases such as systemic lupus erythematosus, BD is not associated with the acceleration of atherosclerosis [10, 11]. Thus, the main mechanisms involved are stenotic lesions and coronary aneurysms, which are the most frequently detected lesions. Also, coronary vasculitis can lead to coronary occlusion by causing intimal fibrous thickening [10]. The compression of the coronary artery by external aneurysmal dilation of the left sinus of Valsalva and also impaired microvascular function are thought to be causes of acute coronary events in this context [12]. The presence of a fresh floating thrombus in angiographically normal coronary arteries has also been reported in the literature suggesting an underlying pro-thrombotic etiology [8].

During BD, two mechanisms can explain thrombosis: hypercoagulable state and vasculitis. The hypercoagulable state is believed to be due to increased platelet aggregation but also to inhibition of fibrinolysis. Increased platelet aggregation is explained by endothelial dysfunction, leading to decreased prostacyclin levels and increased production of von Willebrand factor and factor VIII [13, 14]. Vasculitis of BD affects all layers of vessels; during the acute phase, it is characterized by the presence of lymphocytic infiltrate and significant fibrous at an advanced stage. Vasculitis remains the main mechanism of thrombosis [9]. For our patient, both vasculitis and hypercoagulable state appeared to be involved in coronary thrombosis.

In the literature, several therapeutic approaches have been defined for managing myocardial infarction (MI) in patients with BD. According to the European Society of Cardiology (ESC) guidelines for the management of acute myocardial infraction in a patient with ST-segment elevation, reperfusion therapy is indicated in all patients within 12 hours of symptom onset and persistent ST-segment elevation, and primary percutaneous coronary intervention (PCI) is the recommended reperfusion strategy over fibrinolysis if performed within 120 minutes of the first medical contact [15]. That’s why some authors suggested that percutaneous coronary dilatation was the most recognized revascularization option during STEMI secondary to BD’s coronary thrombosis [16]. Cetin et al. proposed that thrombus aspiration and administration of GPII b III a inhibitors should be used first, ballooning and/or stenting should be selected as the second choice on account of pathergy like effect [17]. But in some cases treating a coronary thrombosis in patients with BD with PCI tends to result in an increased rate of stent thrombosis and thrombosis recurrence during long-term follow-up and carries a high possibility for the development of coronary artery aneurysm [18]. Therefore, Tsuboi et al. recommended that it is important to administer immunosuppressive drugs, in addition to antithrombotic drugs, such as warfarin, before and after PCI in vasculo-BD to prevent relapse or exacerbation. Corticosteroids, colchicine, azathioprine, and cyclophosphamide were the most commonly used agents in previous reports [19]. Alternatively, another approach is the administration of thrombolytic therapy at the early hours of MI. Kosar et al. reported a successful case of fibrinolysis in a patient with BD [20]. 

Some authors report using only a high dose of corticosteroids to treat some cases of MI in patients with BD. Hattori and Kawana reported a case of a patient treated using sodium methylprednisolone succinate at a dose of 1000 mg for three days, followed by oral administration of prednisolone at a dose of 60 mg/day, leading to improvement of all the symptoms after three weeks and normalization of the electrocardiogram findings [18]. Finally, the therapeutic approach remains complex and varies as to the underlying pathology.

In our case, the initial sole antiaggregant, anticoagulant treatments, and thrombus aspiration contributed to the recanalization of coronary thrombosis and the improvement of cardiac ischemia. The patient also received dual antiplatelet therapy (acetylsalicylate and clopidogrel), antivitamin K (Sintrom), bisoprolol, ramipril, and atorvastatin. As the diagnosis of Behcet's disease was only suspected, we chose to start a treatment combining corticosteroids and immunosuppressants after establishing the diagnosis according to international criteria. The clinical course was good, the patient did not develop heart failure signs, and repeated echocardiography showed improved left ventricular ejection fraction at 55%.

## Conclusions

Although coronary artery thrombosis, as a cardiac involvement, is a rare manifestation of BD, but may worsen the prognosis of this disease and should be kept in mind, especially in young patients presenting with chest pain accompanied by systemic involvement. The therapeutic approach for this complication remains complex. More research is needed for a better understanding and management of this complication.
